# Analysis of Clinical Features in Children with Vasovagal Syncope Complicated by Convulsions or Incontinence

**DOI:** 10.3390/biomedicines14010036

**Published:** 2025-12-23

**Authors:** Wenrui Xu, Chunyu Zhang, Junbao Du, Hongfang Jin, Ying Liao

**Affiliations:** 1 Department of Pediatrics, Peking University First Hospital, Beijing 100034, China; xuwenrui@pku.edu.cn (W.X.); chunyudoctor@pku.edu.cn (C.Z.); drjunbaodu@pku.edu.cn (J.D.); 2 State Key Laboratory of Vascular Homeostasis and Remodeling, Peking University, Beijing 100191, China

**Keywords:** vasovagal syncope, children, convulsions or incontinence, characteristics

## Abstract

**Objective**: Vasovagal syncope (VVS) complicated by convulsions or incontinence (atypical VVS) has distinct manifestations prone to misdiagnosis. This study sought to investigate the clinical manifestations and contributing risk factors of atypical VVS in pediatric patients, with the goals of providing a scientific basis for early identification and improving diagnostic accuracy. **Methods**: We carried out a case–control study focusing on children with a diagnosis of VVS who received inpatient care in the Pediatric Department of Peking University First Hospital from January 2021 to June 2025. Patients who experienced convulsions or incontinence during syncopal episodes were assigned to the atypical VVS group, while those without these symptoms formed the control group. The clinical data of the two groups were compared, and logistic regression analysis was utilized to detect factors associated with atypical VVS. **Results**: A total of 393 qualified patients were recruited; there were 68 cases in the atypical VVS group and 325 cases in the control group. The age of the first syncopal episode in children with atypical VVS was significantly lower than that in the control group [9.5 (7.0, 12.0) vs. 11.0 (8.0, 13.0) years, *p* < 0.05]. Additionally, the atypical VVS group showed higher rates of syncope-related trauma (22.1% vs. 9.2%, χ^2^ = 7.905, *p* < 0.01), positive syncope-related family history (35.3% vs. 22.8%, χ^2^ = −4.067, *p* < 0.05), and syncope triggered by central factors (33.8% vs. 19.7%, χ^2^ = 5.721, *p* < 0.05). The Holter monitoring results revealed that the minimum heart rate was significantly reduced in the atypical VVS group [48.0 (44.8, 52.0) vs. 50.0 (47.0, 54.0) beats/min, *p* < 0.01]. The analysis of heart rate variability (HRV) showed that the index of the percentage of adjacent normal-to-normal interval differences greater than 50 ms [pNN50; 23.4 (16.6, 34.2) vs. 20.1 (13.1, 28.4), *p* < 0.05)] and the root mean square of successive differences between adjacent normal cycles [rMSSD; 47.5 (41.0, 64.0) vs. 45.0 (36.0, 56.0), *p* < 0.05)] was significantly higher in the atypical VVS group than in the control group. Two independent factors associated with atypical VVS were detected with multivariate logistic regression: age at the first episode (OR = 0.874, 95% CI 0.802–0.952, *p* < 0.01) and minimum heart rate (OR = 0.921, 95% CI 0.879–0.965, *p* < 0.01). **Conclusions**: Pediatric patients with atypical VVS present with lower minimum heart rate and a higher incidence of syncope induced by central triggers. Compared with children with typical VVS, those with atypical VVS exhibit more pronounced autonomic nervous system imbalance, characterized by enhanced vagal tone. For children with VVS showing these clinical features, careful differential diagnosis, close follow-up, and vigilance against prolonged asystole during syncopal episodes are recommended.

## 1. Introduction

Vasovagal syncope (VVS) is the most common etiology for syncope in children, comprising 60–80% of all syncopal occurrences. The typical clinical presentation of VVS includes transient loss of consciousness with concurrent limb flaccidity, without convulsions or urinary/fecal incontinence. Episodes are self-limiting and do not leave residual sequelae. However, some children with VVS may display atypical symptoms during syncopal attacks, such as convulsive movements or urinary/fecal incontinence—manifestations that often lead to misdiagnosis as epilepsy or cardiogenic syncope [1,2,3]. Compared with typical VVS, which is characterized solely by limb flaccidity during episodes, the co-occurrence of convulsions and incontinence may indicate more significant transient cerebral dysfunction secondary to inadequate cerebral perfusion, with an elevated risk of syncope-related trauma. Moreover, the presence of convulsions and incontinence can impose significant psychological distress on affected children and their parents, potentially impacting the child’s quality of life [4,5]. Given these challenges, investigating the clinical features of atypical VVS in children is essential to enabling early identification and accurate diagnosis. Such research can also facilitate the development of targeted therapeutic interventions, alleviate the psychological burden on patients and families, and provide insights into the underlying pathogenesis of VVS. This study performed a retrospective review of the clinical data of children with VVS complicated by convulsions or incontinence (atypical VVS) hospitalized in the Pediatric Department of Peking University First Hospital from January 2021 to June 2025. Through a case–control study design, we aimed to identify the risk factors for atypical VVS in children, delineate its clinical characteristics, and thus establish a scientific basis for the early identification of such atypical cases.

## 2. Materials and Methods

### 2.1. Study Participants

We adopted a case–control study approach; the inclusion criteria were defined as follows: (1) children aged between 0 and 18 years hospitalized in Peking University First Hospital’s Pediatric Department between January 2021 and June 2025 and (2) confirmed diagnosis of VVS [6].

#### 2.1.1. Inclusion Criteria for VVS

We established the diagnosis of VVS according to the guideline [6]: (1) higher prevalence in older children; (2) common triggers including prolonged standing, swift postural change from supine or squatting stance to an upright stance, mental stress, or crowded/muggy environments; (3) presence of syncopal episodes; (4) positive findings on head-up tilt test (HUTT); (5) exclusion of other etiologies. A positive HUTT was established based on the development of syncope or presyncope during the testing process accompanied by any of the following: (1) hypotension (a systolic blood pressure [SBP] of ≤80 mmHg, a diastolic blood pressure [DBP] of ≤50 mmHg, or a reduction of ≥25% in mean arterial pressure); (2) bradycardia (for 4–6-year-olds, heart rate [HR] < 75 beats/min; for 7–8-year-olds, <65 beats/min; and for children aged > 8 years, <60 beats/min); (3) the occurrence of sinus arrest or junctional escape rhythm; (4) atrioventricular block of second degree or higher, or asystole lasting ≥ 3 s. VVS subtypes were categorized as follows: vasodepressor type (significant hypotension in the absence of notable bradycardia), cardioinhibitory type (predominant bradycardia that is not accompanied by significant hypotension), and mixed type (significant reductions in both blood pressure and heart rate).

#### 2.1.2. Exclusion Criteria for Other Etiologies

To ensure the accuracy of the etiological diagnosis of VVS, all enrolled children underwent a comprehensive battery of auxiliary examinations, including echocardiography, 12-lead standard electrocardiogram (ECG), 24 h ambulatory ECG, myocardial enzyme tests, video electroencephalography (vEEG), and cranial imaging [cranial computed tomography (CT) or magnetic resonance imaging (MRI)]. Patients with potential other causes of syncope or alternative diagnosis presenting with syncope-like events were excluded based on the results of these evaluations, with specific exclusion criteria as follows: (1) Regarding neurological diseases, patients were excluded if any of the following conditions were present: ① history of perinatal hypoxia–ischemia, craniocerebral trauma, or other neurological disorders potentially associated with seizures; ② vEEG showing epileptiform discharges or abnormal background activity; ③ cranial imaging findings indicating intracranial structural abnormalities (e.g., space-occupying lesions and congenital malformations). (2) Regarding cardiogenic syncope, patients were excluded if any of the following conditions were confirmed: ① detection of malignant arrhythmias (e.g., ventricular tachycardia, ventricular fibrillation, atrial flutter with rapid ventricular response, atrial fibrillation, Wolff–Parkinson–White syndrome complicated by atrial fibrillation, and high-grade atrioventricular block) based on 12-lead standard ECG or 24 h ambulatory ECG; ② elevated myocardial enzyme levels suggesting myocardial injury; ③ clinical or genetic evidence of cardiac ion channelopathies (e.g., long QT syndrome, short QT syndrome, Brugada syndrome, and catecholaminergic polymorphic ventricular tachycardia); ④ echocardiographic findings revealing cardiac structural or functional abnormalities (e.g., complex congenital heart disease, cardiomyopathy, pulmonary arterial hypertension, aortic stenosis, and heart failure). (3) Regarding incomplete clinical data, patients were excluded if clinical manifestations, laboratory results (including myocardial enzyme tests), or imaging data were insufficient to conduct a comprehensive etiological assessment.

Participants were categorized into two groups. The atypical VVS group comprised pediatric patients with VVS who exhibited limb convulsions and/or urinary/fecal incontinence during at least one syncopal episode. We further recruited children with VVS who were free of these manifestations and received treatment during the same study period to serve as the control group.

Approval for this study was obtained from the Ethics Committee of Peking University First Hospital (Approval No. 2025R0326; date of approval: 28 August 2025), with the study protocol being exempt from informed consent.

### 2.2. Study Methods

Clinical data-related information was obtained from the hospital’s electronic medical record system, with details including the following: (1) Demographic profiles and baseline measurements encompassed age, gender, body weight, height, body mass index (BMI), baseline HR, SBP and DBP, age at the first syncopal episode, frequency of syncopal episodes, syncope triggers, prodromal symptoms, occurrence of trauma associated with syncope, and a family history of similar conditions. Syncope triggers were categorized into peripheral and central types, where among the former were postural changes, prolonged standing, and rest following exercise, while central triggers included intramuscular injection, venous blood sampling, emotional stress, and painful stimuli. (2) The following auxiliary examination findings were also obtained: ① routine laboratory analyses, encompassing a complete blood count, renal function assessments, liver function tests, and myocardial enzyme panels; ② hemodynamic subtypes identified with the HUTT; ③ results of 24 h ambulatory electrocardiography and echocardiography; ④ the indicators of VVS susceptibility of flow-mediated vasodilation (FMD), volume of 24 h urine, and 24 h urinary sodium content; ⑤ heart rate variability (HRV) parameters in the time domain, i.e., the root mean square of successive differences between adjacent normal cycles (rMSSD), the standard deviation of 5 min average normal-to-normal intervals (SDANN), the standard deviation of all normal-to-normal intervals (SDNN), and the percentage of adjacent normal-to-normal interval differences over 50 ms (pNN50), and in the frequency domain, i.e., low frequency (LF), high frequency (HF), and the ratio of LF to HF (LF/HF). The HRV index was obtained using 24 h Holter monitors (Mortara, Milwaukee, WI, USA), and subsequent HRV analysis was performed with a dedicated analyzer (H-Scribe 7.0; Mortara, Milwaukee, WI, USA). RMSSD, SDANN, SDNN, LF, and HF were recorded in milliseconds (ms), while pNN50 was recorded as a percentage value (%).

### 2.3. Statistical Analysis

SPSS 26.0 and R Studio software (version 2025.05.1 Build 513) were utilized for data analysis. For the categorical variables in this study, data were presented in the form of counts, and the continuous variables that conformed to a normal distribution according to the Shapiro–Wilk test were depicted as means ± standard deviation. To assess differences in these variables between the two groups, the independent samples *t*-test was adopted. Regarding continuous variables that did not conform to a normal distribution, they were presented as medians (25th, 75th percentiles), and intergroup comparisons were carried out with the Mann–Whitney U test. Comparisons of proportions were assessed using either Fisher’s exact test or the chi-square test, with the choice of test being determined by suitability. For intergroup comparisons of age at the first episode, further age-stratified analysis was performed. To mitigate the potential confounding effects of age and gender on heart rate-related indices and HRV parameters, we employed analysis of covariance (ANCOVA) to systematically compare intergroup differences in these autonomic and cardiac metrics between the atypical VVS group and the control group. Age and gender were incorporated as key covariates in the statistical model to adjust for their potential confounding impacts on the primary outcomes of interest.

A three-stage statistical strategy (“dimensionality reduction screening—association validation—independent effect analysis”) was adopted in this study: First, Lasso logistic regression with 10-fold cross-validation was used for the reduction in the dimensionality of the candidate variables. These variables included demographic data (gender, age, HR, SBP, and DBP), clinical history-related factors (age of syncope onset, disease duration, number of syncopal episodes, type of precipitating factors, and family history of syncope), 24 h ambulatory electrocardiogram parameters (total heartbeats, minimum HR, average HR, and maximum HR), and HRV indices (SDNN, SDANN, pNN50, rMSSD, HF, LF, and LF/HF ratio). Variables with non-zero coefficients were retained based on the optimal λ value (λ.min). Subsequently, univariate regression analysis was performed on the screened variables, and the Benjamini–Hochberg method was used for false discovery rate (FDR) correction to control for false positives arising from multiple testing. Considering clinical research conventions and the necessity of controlling for confounding factors, the age and gender variables were forcefully included in the analysis. Variables to be incorporated into the multivariate model were ultimately determined by combining the gender variable with the criterion of “FDR-adjusted *p* < 0.1”. Finally, eligible variables were included in the multivariate regression model to analyze the independent association between each variable and the outcome. Adjusted odds ratios (ORs) with 95% confidence intervals (CIs) or regression coefficients (β) with 95% CIs, as well as *p*-values, were calculated. Statistical significance was defined as a two-tailed *p*-value < 0.05.

## 3. Results

### 3.1. Decreased BMI in Atypical VVS Pediatric Cases Versus the Control Group

**Figure 1:**
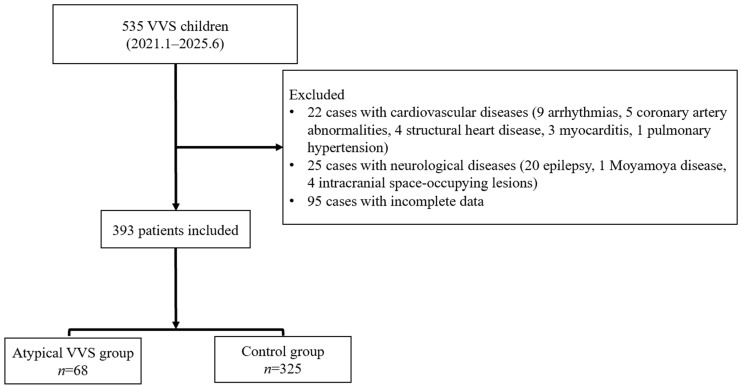
Flowchart of enrolled study participants. VVS, vasovagal syncope.

From January 2021 to June 2025, a cumulative number of 535 children with a diagnosis of VVS were admitted to Peking University First Hospital’s Pediatric Department. Following the implementation of the established inclusion and exclusion standards, 393 cases were finally included in the study (Figure 1), among which 68 (17.3% of the total) were classified as atypical VVS. Of the children with atypical VVS (*n* = 68), there were 30 boys and 38 girls, and their median age at presentation was 12.0 years. The control group, meanwhile, comprised 325 children (201 girls and 124 boys), with a median age at presentation of 12.0 years. No statistically significant differences were identified when comparing the two groups with respect to height, weight, baseline HR, baseline SBP, or baseline DBP. In contrast, BMI was lower in children with atypical VVS relative to the controls [17.5 (15.4, 19.9) vs. 18.7 (16.6, 21.4) kg/m^2^, *p* < 0.05] (Table 1).

### 3.2. Children with Atypical VVS Have a Higher Proportion of Syncope-Related Trauma and More Frequent Central Triggers

All children presented with an episodic course. Among the 68 children in the atypical VVS group, 59 experienced limb convulsions during at least one syncopal episode, 20 had urinary/fecal incontinence, and 11 exhibited both limb convulsions and incontinence. No abnormal physical signs were identified in the physical examination of all children.

**Figure 2:**
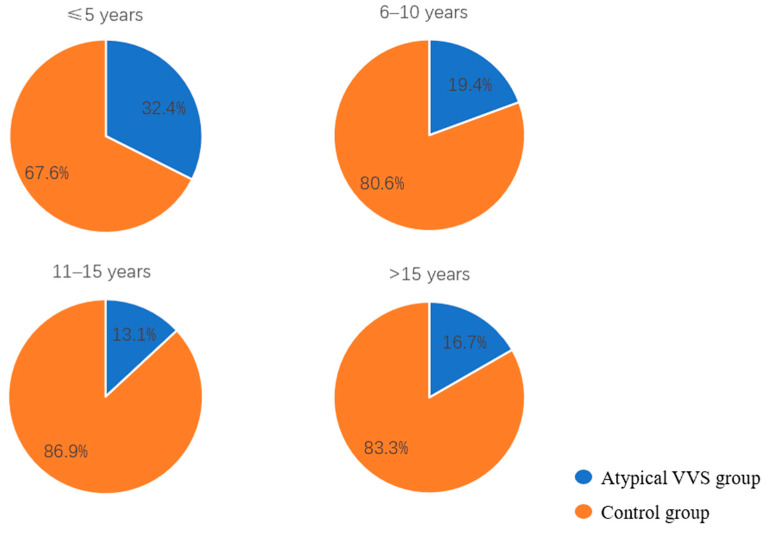
Proportions of children in the atypical VVS and control groups stratified by age at the first syncopal episode.

The age of first syncopal episode in children with atypical VVS was significantly younger than that in the control group [9.5 (7.0, 12.0) vs. 11.0 (8.0, 13.0) years, *p* < 0.05]. Given that VVS pathogenesis is linked to autonomic nervous system function and based on the developmental stages of children and characteristics of autonomic nervous system maturation in pediatric populations reported in previous studies [7,8], the age at the first episode was stratified into four categories: 0 to 5 years, 6 to 10 years, 11 to 15 years, and greater than 15 years. Further stratified analysis revealed that the proportion of children with the first episode at 0–5 years was significant higher in the atypical VVS group as opposed to the control group (17.6% vs. 7.7%, χ^2^ = 6.534, *p* < 0.05), with a decreasing trend in the proportion of atypical VVS cases with the increase in age (Figure 2). The proportion of syncope-related trauma was higher in the atypical VVS group (22.1% vs. 9.2%, χ^2^ = 7.905, *p* < 0.01). In cases with syncope-related trauma, only one child had a skull fracture, while the rest suffered from facial or limb abrasions. In addition, the proportion of patients with a positive syncope-related family history in the atypical VVS group was higher than that in the control group (35.3% vs. 22.8%, χ^2^ = −4.067, *p* < 0.05). No statistically significant disparities in disease duration or number of syncopal episodes were found when comparing the two groups (Table 2).

Regarding syncope triggers, the most common trigger in the atypical VVS group was prolonged standing (64.7%), followed by postural changes (38.2%) and painful stimuli (19.1%). Other triggers included emotional stress (13.2%), intramuscular injection or venous blood sampling (11.8%), and upright rest after exercise (10.3%). In the control group, the most frequent trigger was prolonged standing (74.8%), followed by postural changes (42.5%) and upright rest after exercise (14.2%). Other triggers included emotional stress (8.9%), intramuscular injection or venous blood sampling (7.1%), and painful stimuli (6.2%). In terms of trigger composition, central triggers were more prevalent in the atypical VVS group than in their control counterparts (33.8% vs. 19.7%, χ^2^ = 5.721, *p* < 0.05) (Table 2).

When surveyed about prodromal symptom occurrence, 88.2% of children with atypical VVS and 94.2% of children in the control group indicated having experienced such symptoms. In both groups, dizziness and amaurosis fugax were the most prevalent prodromal symptoms (Table 2).

### 3.3. Heart Rate Variability and Hemodynamic Characteristics in Children with Atypical VVS

No significant disparities in 24 h urinary sodium excretion, 24 h urine output, or FMD existed between the two groups (Table 3).

The Holter monitoring results revealed that the minimum HR was significantly reduced in the atypical VVS group [48.0 (44.8, 52.0) vs. 50.0 (47.0, 54.0) beats/min, *p* < 0.01]. According to the HRV analysis, rMSSD [47.5 (41.0, 64.0) vs. 45.0 (36.0, 56.0), *p* < 0.05)] and pNN50 [23.4 (16.6, 34.2) vs. 20.1 (13.1, 28.4), *p* < 0.05)] showed a statistically significant elevation in the atypical VVS group as opposed to the control group, while no differences were found in SDNN, SDANN, HF, LF, or LF/HF between the two groups (Table 3).

To mitigate the confounding influences of age and gender on heart rate-related indices and HRV parameters, we conducted an analysis of covariance to examine intergroup differences in these metrics, with age and gender included as covariates. The findings revealed that following adjustment for age and gender, statistically significant intergroup disparities persisted in minimum HR, average HR, rMSSD, pNN50, HF, and LF (Table 4).

Among the children with atypical VVS, a higher proportion presented with cardioinhibitory or mixed hemodynamic subtype (14.7% vs. 7.7%), though this difference failed to achieve statistical significance. Notably, the atypical group had a significantly higher incidence of malignant VVS episodes (defined as VVS with ≥3 s asystole) than the control group (7.4% vs. 0.6%, *p* < 0.01).

### 3.4. Age at the First Episode and Minimal Heart Rate as Independent Correlates of Atypical VVS

**Figure 3:**
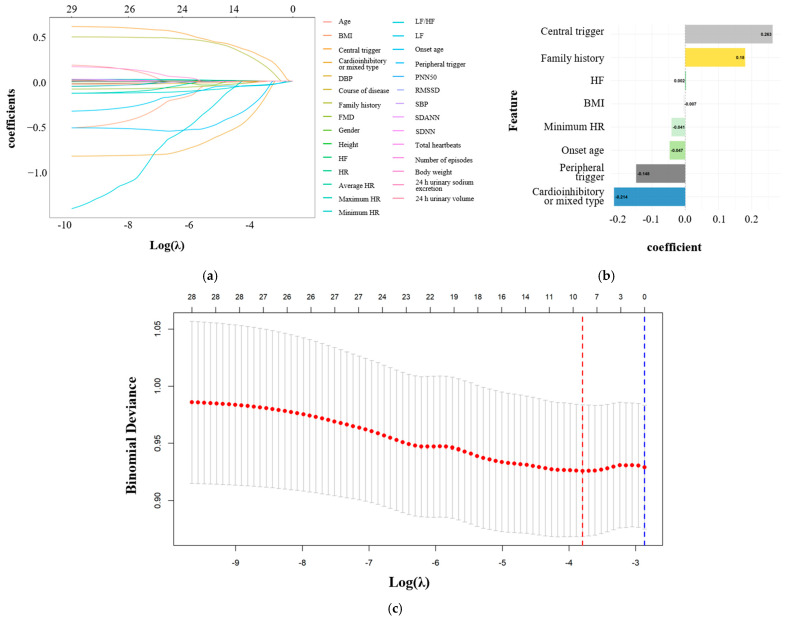
Factors selected in Lasso regression for identifying independent correlates of atypical VVS. (**a**) Lasso coefficient profile plot. The regression coefficient of each independent variable changes with variations in λ, where colored lines represent distinct variables. As λ increases, the model shows a higher degree of compression. (**b**) Lasso coefficient plot for variable selection, illustrating the magnitude and direction of regression coefficients associated with the selected features. A positive coefficient denotes a positive correlation between the feature and the dependent variable, whereas a negative coefficient indicates a negative correlation; further, the greater the absolute value of the coefficient, the more substantial the influence of the corresponding feature on the model. (**c**) Cross-validation plot for the penalty term. The left vertical line (red line) corresponds to the λ value that produces the minimum mean squared error (MSE), whereas the right vertical line (blue line) corresponds to the λ value associated with one standard error from the minimum MSE. For this study, the optimal λ value was determined by selecting the left vertical line. BMI, body mass index; DBP, diastolic blood pressure; FMD, flow-mediated vasodilation; HF, high frequency; HR, heart rate; LF, low frequency; pNN50, percentage of adjacent normal-to-normal interval differences greater than 50 ms; rMSSD, root mean square of successive differences between adjacent normal cycles; SBP, systolic blood pressure; SDANN, standard deviation of the 5 min average normal-to-normal intervals; SDNN, standard deviation of all normal-to-normal intervals.

Lasso regression analysis was performed with “atypical VVS status” as the dependent variable, with eight candidate parameters identified and selected based on the preliminary analysis results. (Figure 3): BMI, age at the first syncopal episode, a positive family history, syncope induced by peripheral triggers, syncope induced by central triggers, minimum heart rate, HF, and hemodynamic classification (cardioinhibitory or mixed type). After FDR correction, seven variables with a *p*-value < 0.1 were ultimately included in the multivariate logistic regression analysis: age at the first syncopal episode, positive family history, syncope induced by peripheral triggers, syncope induced by central triggers, minimum heart rate, HF, and hemodynamic classification (Table 5). Additionally, gender and age were also included in the multivariate logistic regression. The collinearity analysis demonstrated that the variance inflation factor (VIF) of all variables was below 10, while their tolerance values exceeded 0.1, thus ruling out significant multicollinearity (Table 6).

According to the multivariate logistic regression analysis, age at the first episode (OR = 0.874, 95% CI 0.802–0.952, *p* = 0.002) and minimal heart rate (OR = 0.921, 95% CI 0.879–0.965, *p* = 0.001) were independent correlates of atypical VVS (Table 5). Specifically, each 1-year reduction in age at the first episode correlated with a 1.14-fold elevated probability of atypical VVS, and each 1-beat/minute decrease in minimal heart rate corresponded to a 1.09-fold increased probability (Table 7).

## 4. Discussion

To date, research on pediatric VVS complicated by convulsive-like movements or incontinence remains extremely limited, and the underlying pathophysiological mechanisms have not been systematically elucidated. Focusing on an atypical VVS subgroup characterized by syncope with either convulsive-like movements or incontinence (accounting for 17.3% of pediatric VVS cases at our center), this study analyzed clinical features, autonomic nervous function, and related risk factors to propose a potential pathophysiological link: children with atypical VVS exhibit increased baseline vagal tone, which may induce severe cardiac inhibition during syncopal episodes, leading to significant cerebral hypoperfusion and ultimately manifesting as atypical symptoms.

The autonomic nervous system serves a pivotal function in the pathogenic mechanism underlying VVS, though its precise role requires further elucidation. Two key regulatory patterns are proposed: progressive sympathetic activation preceding syncope, usually linked to the Bezold–Jarisch reflex, and sympathetic inhibition with vagal dominance, usually associated with baroreflex dysfunction or M_2_ muscarinic receptor overexpression [9,10,11]. HRV assesses autonomic function, pNN50 and rMSSD reflect parasympathetic tone and HF components vagal tone, and LF components are dually regulated by both sympathetic and parasympathetic nerves [12,13,14,15]. Our study results show that rMSSD and pNN50 were significantly higher in children with atypical VVS than in the control group; after adjusting for age and gender, rMSSD, pNN50, HF, and LF in the atypical VVS group were all significantly elevated compared with the control group. These findings suggest that children with atypical VVS exhibit more pronounced autonomic dysfunction characterized by parasympathetic predominance, and increased vagal tone may represent its core pathophysiological basis.

Notably, increased vagal tone may induce cardiac inhibition: children in the atypical VVS group exhibited a significantly lower minimum HR than those in the control group, with a higher proportion of malignant VVS (defined as syncope accompanied by severe bradycardia or asystole during episodes). Severe cardiac inhibition further reduces cardiac output, leading to significant cerebral hypoperfusion—this link may serve as the key bridge connecting autonomic dysfunction to atypical symptoms. Multiple studies have confirmed that abnormal vagal excitation can inhibit the cardiac sinoatrial and atrioventricular nodes, resulting in bradycardia and reduced cardiac output. This lowers global cerebral blood perfusion below the threshold required to maintain normal neural function, exacerbating acute cerebral ischemia–hypoxia [10,16,17]. The cerebral cortex has a high metabolic rate and limited energy reserves, rendering it highly sensitive to ischemia–hypoxia caused by local microcirculatory disruption—this mechanism is a crucial trigger for abnormal manifestations such as convulsive-like movements [18].

Urinary incontinence is closely associated with impaired regulatory function of the higher brain centers over the spinal micturition center. Under normal urine storage conditions, the higher brain centers maintain detrusor muscle relaxation and urethral sphincter contraction by inhibiting parasympathetic activation mediated by the spinal micturition center. When this regulatory pathway is disrupted by factors such as cerebral hypoperfusion, it may trigger involuntary detrusor contraction and urethral sphincter relaxation, ultimately leading to urinary incontinence [19,20,21]. In children, cerebral autoregulatory function is not yet fully developed, resulting in weak compensatory capacity against pathophysiological perturbations such as autonomic dysfunction and external stimuli [22]. When abnormal vagal excitation induces severe hemodynamic fluctuations, their limited cerebral perfusion reserve is insufficient to offset the sudden drop in perfusion pressure, making it more likely to breach the minimum threshold for maintaining cerebral blood flow. This leads to severe cerebral hypoperfusion, which in turn increases the risk of atypical manifestations such as convulsive-like movements and urinary incontinence.

In the present study, the incidence of atypical VVS (17.3%) was significantly higher than the reported incidence of convulsive-like movements during the head-up tilt test (HUTT) in pediatric VVS patients from previous single-center studies (1.9%~3.0%) [23,24,25]. This discrepancy is primarily attributed to differences in study design: previous studies only documented manifestations during the HUTT, whereas the current study included all syncopal episodes throughout the natural course of the disease. This suggests that a single HUTT may not adequately simulate the triggers and severity of syncope in real-world settings, emphasizing the need for detailed medical history taking in clinical practice to avoid the misdiagnosis of this atypical subgroup.

Incontinence is rarely reported in pediatric VVS. A study by Zhang Q. et al. [9] showed that the incidence of urinary incontinence in patients with cardiogenic syncope was approximately 56%, while in the present study, the incidence was 5.1% in the enrolled VVS children. Although significantly lower than that in cardiogenic syncope, this rate remains non-negligible—indicating that urinary incontinence is not a specific manifestation of cardiogenic syncope. Its occurrence may reflect the severity of cerebral hypoperfusion; therefore, when encountering patients with syncope complicated by urinary incontinence in clinical practice, a comprehensive cardiac evaluation is necessary to rule out organic heart disease.

Additionally, epidemiological data on syncope-related trauma in pediatric VVS populations are scarce. Adult studies have reported that 23%~33% of VVS patients experience syncope-related trauma, with 13.9% being severe injuries (e.g., fractures and craniocerebral trauma) [26,27,28]. Data from our center showed that the incidence of syncope-related trauma in pediatric VVS patients was approximately 11.5% and children with atypical VVS had a significantly higher risk of trauma. This may be related to the sudden onset of the events or convulsive-like movements, which impairs the patients’ ability to protect themselves during syncope. Thus, more aggressive interventions and close follow-up are warranted for this subgroup to minimize trauma risk.

The multivariate analysis revealed that a younger age at the first syncopal episode was an independent correlate of atypical VVS. The proportion of children with onset at 0–5 years of age was significantly higher in the atypical VVS group than in the control group (17.6% vs. 7.7%, χ^2^ = 6.534, *p* = 0.011), indicating that young children are a high-risk population for atypical VVS. The underlying mechanism may be associated with the heterogeneity of autonomic nervous system development and pathological dysfunction. Previous studies have demonstrated that the maturation of pediatric autonomic function is age-dependent: HRV-related parameters peak during adolescence, and the autonomic balance in healthy young children is inherently dominated by sympathetic activity [29,30]. In contrast, our study identified two distinct characteristics in children with atypical VVS: “an earlier age of onset” and “more pronounced vagal dominance” than age-matched controls. Given the early onset age and higher rate of positive family history in this subgroup, we hypothesize that the genetic background may be a key contributing factor to the early autonomic nervous system imbalance observed in these children. This imbalance could lead to the abnormal elevation of vagal tone, ultimately resulting in syncope accompanied by atypical symptoms. However, further genetic analyses are required to validate this inference.

Regarding the diagnosis and treatment algorithm for children with syncope, when admitting young children with syncope, convulsions and incontinence are more likely to be features supporting atypical VVS, while older children with similar manifestations require more thorough evaluation to rule out other etiologies. Given that such children more frequently present with malignant VVS, enhanced monitoring is recommended during the HUTT to alert against prolonged asystole and potential trauma induced by convulsions. Regarding treatment, for children presenting with concurrent convulsions and incontinence, greater emphasis is placed on avoiding central triggers and intensifying monitoring during exposure to such triggers to prevent trauma. Medications that may reduce sympathetic tone and further exacerbate the vagal-predominant autonomic imbalance (e.g., β-blockers) may be inappropriate in their management. Zou et al. [24] reported that the prognosis of pediatric VVS patients with convulsive-like movements during the HUTT was comparable to that of those without convulsive manifestations, but the study had a short follow-up period (with a median follow-up of 4 months). Further research is still needed to clarify the long-term prognosis of this subgroup.

This study has several limitations. Firstly, the single-center retrospective design resulted in a limited sample size, which may have introduced selection bias and reduced statistical power. Secondly, the assessment of convulsive-like movements and incontinence relied on historical data, lacking objective and unified symptom recording standards. These two symptoms reflect abnormalities of different physiological systems, and their combined analysis may obscure potential differences in underlying mechanisms. Although between-group differences in HR and HRV were statistically significant, their absolute values were small, requiring cautious interpretation of clinical relevance. Adding time-phased assessments in subsequent analyses could clarify if circadian rhythm differences affect these small variations, further enhancing the accuracy of the conclusion. In the future, we also plan to perform HRV analysis during the head-up tilt test to clarify the dynamic changes in autonomic regulation during orthostasis and before/after syncopal events. Furthermore, the study results have not been validated on external independent samples. Future studies should adopt multi-center, prospective designs with large sample sizes, stratify participants by specific symptoms, standardize symptom assessment criteria, supplement long-term follow-up data, and optimize conclusions through external validation. This will enable a more accurate elucidation of the clinical significance and prognostic value of different atypical VVS manifestations.

## 5. Conclusions

In summary, this study systematically delineates the unique clinical profiles of pediatric atypical VVS, characterized by an earlier age of onset, a higher prevalence of central trigger-induced episodes, an elevated risk of syncope-related trauma, and prominent vagal tone dominance. These findings provide valuable insights for the clinical recognition of this distinct subgroup. Additionally, convulsive-like movements and urinary incontinence are not specific to epilepsy or cardiogenic syncope; their occurrence in atypical VVS may signal severe cardioinhibitory responses. Notably, while the current results provide preliminary guidance for clinicians to identify high-risk patients based on onset age, hemodynamic phenotypes, accompanying symptoms, and HRV features, substantial additional research is imperative before these findings can reliably inform clinical decision making. Future investigations should validate these observations in large-scale multi-center prospective cohorts, standardize the assessment of atypical symptoms, and integrate long-term follow-up data to clarify the prognostic significance of the identified risk factors.

## Figures and Tables

**Table 1:** Comparison of baseline characteristics between the atypical VVS group and control group in pediatric patients.

	Atypical VVS Group (N = 68)	Control Group (N = 325)	t/Z/χ^2^ Value	*p*-Value
Age, year	12.0 (9.0, 14.0)	12.0 (11.0, 14.0)	1.248	0.209 ^a^
Gender			0.608	0.436 ^b^
Male, *n* (%)	30 (44.1)	124 (38.2)	-	-
Female, *n* (%)	38 (55.9)	201 (61.8)	-	-
Height, cm	160.0 (150.0, 167.0)	160.0 (140.8, 167.5)	0.497	0.619 ^a^
Body weight, kg	48.4 (38.5, 57.2)	45.9 (31.4, 54.6)	1.501	0.1335 ^a^
BMI, kg/cm^2^	17.5 (15.4, 19.9)	18.7 (16.6, 21.4)	2.116	0.034 ^a^
Baseline SBP, mmHg	112.0 (106.0, 120.0)	112.0 (105.3, 117.3)	0.930	0.353 ^a^
Baseline DBP, mmHg	67.5 ± 8.3	65.9 ± 8.3	1.469	0.142 ^c^
Baseline HR, beats/min	82.5 (78.8, 91.3)	88.0 (79.0, 94.0)	1.152	0.249 ^a^

BMI, body mass index; DBP, diastolic blood pressure; HR, heart rate; SBP, systolic blood pressure; VVS vasovagal syncope. ^a^ Result of a Mann–Whitney U test presented as a Z value. ^b^ Result of a chi-square test reported as a χ^2^ value. ^c^ Result of an independent samples *t*-test expressed as a t value.

**Table 2:** Comparison of clinical manifestations between children in the atypical VVS group and the control group.

	Atypical VVS Group (N = 68)	Control Group (N = 325)	Z/χ^2^ Value	*p* Value
Age at first syncopal episode, year	9.5 (7.0, 12.0)	11.0 (8.0, 13.0)	2.227	0.025 ^a^
Age groups by first-episode age				0.027 ^b^
≤5 years, *n* (%)	12 (17.6)	25 (7.7)	6.534	0.011 ^c^
6–10 years, *n* (%)	28 (43.1)	116 (35.7)	0.729	0.393 ^c^
11–15 years, *n* (%)	27 (39.7)	179 (55.1)	5.327	0.021 ^c^
>15 years, *n* (%)	1 (1.5)	5 (1.5)		1.000 ^b^
Course of disease, months	12.0 (1.9, 36.3)	8.0 (1.0, 24.0)	−1.397	0.162 ^a^
Number of episodes	3 (2, 4)	3 (2, 5)	0.039	0.969 ^a^
Syncope-related trauma, *n* (%)	15 (22.1)	30 (9.2)	7.905	0.005 ^c^
Positive family history, *n* (%)	24 (35.3)	74 (22.8)	−4.067	0.044 ^c^
Predisposing factors				
Peripheral triggers, *n* (%)	58 (85.3)	300 (92.3)	2.600	0.107 ^c^
Prolonged standing, *n* (%)	44 (64.7)	202 (74.8)	0.066	0.797 ^c^
Postural change, *n* (%)	26 (38.2)	138 (42.5)	0.258	0.612 ^c^
Rest after exercise, *n* (%)	7 (10.3)	46 (14.2)	0.425	0.514 ^c^
Central triggers, *n* (%)	23 (33.8)	64 (19.7)	5.721	0.017 ^c^
Intramuscular injection or venipuncture, *n* (%)	8 (11.8)	23 (7.1)	1.117	0.291 ^c^
Emotion, *n* (%)	9 (13.2)	29 (8.9)	0.754	0.385 ^c^
Pain, *n* (%)	13 (19.1)	20 (6.2)	10.659	0.001 ^c^
Premonitory symptom				
Dizziness, *n* (%)	35 (51.5)	219 (67.4)	5.553	0.018 ^c^
Amaurosis fugax, *n* (%)	28 (41.2)	190 (58.5)	6.120	0.013 ^c^
Blurred vision, *n* (%)	20 (29.4)	70 (21.5)	1.554	0.213 ^c^
Tinnitus, *n* (%)	4 (5.9)	30 (9.2)	0.430	0.512 ^c^
Gastrointestinal symptoms, *n* (%)	19 (27.9)	86 (26.5)	0.010	0.920 ^c^
Palpation, *n* (%)	5 (7.4)	50 (15.4)	2.383	0.123 ^c^
Absent, *n* (%)	8 (11.8)	19 (5.8)		0.109 ^b^

VVS, vasovagal syncope. ^a^ Result of a Mann–Whitney U test presented as a Z value. ^b^ Result of Fisher’s exact test. ^c^ Result of a chi-square test reported as a χ^2^ value.

**Table 3:** Comparison of ancillary test data between children in the atypical VVS group and the control group.

	Atypical VVS Group (N = 68)	Control Group (N = 325)	t/Z/χ^2^ Value	*p*-Value
24 h urinary sodium excretion, mmol/24 h	115.1 (91.6, 152.0)	119.5 (84.0, 158.4)	0.259	0.796 ^a^
24 h urinary volume, mL	1350.0 (1033.8, 1831.3)	1350.0 (1000.0, 1810.0)	−0.558	0.577 ^a^
FMD, %	10.7 (9.8, 12.7)	11.5 (10.0, 13.7)	1.256	0.208 ^a^
24 h total heartbeats, beats	113,250.7 ± 12,857.9	115,112.4 ± 12,210.1	1.133	0.258 ^b^
Minimum heart rate, beats/min	48.0 (44.8, 52.0)	50.0 (47.0, 54.0)	2.668	0.008 ^a^
Maximum heart rate, beats/min	151.5 (143.5, 159.0)	151.0 (141.0, 159.0)	−0.046	0.964 ^a^
Average heart rate, beats/min	79.0 (73.0, 85.3)	82.0 (75.0, 86.0)	1.290	0.197 ^a^
SDNN, ms	150.5 (135.0, 179.0)	151.0 (129.0, 171.0)	−1.088	0.277 ^a^
SDANN, ms	133.0 (114.8, 157.3)	132.0 (113.0, 151.0)	−0.849	0.396 ^a^
rMSSD, ms	47.5 (41.0, 64.0)	45.0 (36.0, 56.0)	−2.074	0.038 ^a^
pNN50, %	23.4 (16.6, 34.2)	20.1 (13.1, 28.4)	−2.318	0.021 ^a^
HF, ms	26.1 (20.5, 36.7)	24.7 (18.5, 34.2)	−1.739	0.082 ^a^
LF, ms	31.3 (25.1, 40.6)	29.4 (24.4, 35.0)	−1.747	0.081 ^a^
LF/HF	1.14 (0.97, 1.29)	1.18 (0.99, 1.37)	1.337	0.181 ^a^
Cardioinhibitory or mixed type, *n* (%)	10 (14.7)	25 (7.7)	2.600	0.107 ^c^
Malignant VVS, *n* (%)	5 (7.4)	2 (0.6)		0.002 ^d^

FMD, flow-mediated vasodilation; HF, high frequency; LF, low frequency; pNN50, percentage of adjacent normal-to-normal interval differences greater than 50 ms; rMSSD, root mean square of successive differences between adjacent normal cycles; SDANN, standard deviation of the 5 min average normal-to-normal intervals; SDNN, standard deviation of all normal-to-normal intervals; VVS, vasovagal syncope. ^a^ Result of a Mann–Whitney U test presented as a Z value. ^b^ Result of a independent samples *t*-test reported as a t value. ^c^ Result of a chi-square test presented as χ^2^ values. ^d^ Result of Fisher’s exact test.

**Table 4:** ANCOVA results of heart rate-related indices and HRV parameters between two groups after adjusting for age and gender.

Parameter	F	B	*p* (Gender)	*p* (Age)	*p* (Group)
24 h total heartbeats	3.081	−2546.439	0.002	0.000	0.080
Minimum heart rate	11.570	−2.620	0.000	0.000	0.001
Maximum heart rate	0.066	−0.467	0.006	0.000	0.797
Average heart rate	5.093	−2.403	0.014	0.000	0.025
SDNN	3.153	7.699	0.001	0.000	0.077
SDANN	1.818	5.628	0.004	0.000	0.178
rMSSD	6.339	5.858	0.033	0.964	0.012
pNN50	5.802	3.451	0.017	0.207	0.016
HF	4.750	3.796	0.401	0.254	0.030
LF	3.977	2.406	0.000	0.095	0.047
LF/HF	1.953	−0.054	0.010	0.000	0.163

HRV, heart rate variability; HF, high frequency; LF, low frequency; pNN50, percentage of adjacent normal-to-normal interval differences greater than 50 ms; rMSSD, root mean square of successive differences between adjacent normal cycles; SDANN, standard deviation of the 5 min average normal-to-normal intervals; SDNN, standard deviation of all normal-to-normal intervals.

**Table 5:** Results of univariate logistic regression analysis and false discovery rate correction.

Variable	OR	95% CI	*p*-Value	False Discovery Rate-Adjusted *p*-Value
BMI	0.944	0.873–1.014	0.127	0.127
Age at first syncopal episode	0.907	0.835–0.983	0.018	0.048
Positive family history	1.85	1.045–3.222	0.032	0.050
Peripheral trigger	0.483	0.226–1.104	0.070	0.080
Central trigger	2.084	1.163–3.668	0.012	0.047
Minimum heart rate	0.937	0.897–0.978	0.003	0.027
HF	1.021	1.002–1.04	0.024	0.066
Cardioinhibitory or mixed type	0.483	0.226–1.104	0.070	0.093

BMI, body mass index; HF, high frequency.

**Table 6:** Pre-logistic regression multicollinearity analysis.

Variable	Tolerance	VIF
Gender	0.906	1.104
Age	0.383	2.613
Age at first syncopal episode, years	0.369	2.712
Positive family history	0.950	1.052
Peripheral trigger	0.890	1.124
Central trigger	0.822	1.216
Minimum heart rate, beats/min	0.677	1.477
HF	0.787	1.271
Cardioinhibitory or mixed type	0.962	1.040

VIF, variance inflation factor.

**Table 7:** Independent correlates of atypical VVS in children.

	B	SE	Wald	*p*	OR (95% CI)
Age at first syncopal episode	−0.135	0.044	9.566	0.002	0.874 (0.802, 0.952)
Minimum heart rate	−0.082	0.024	12.002	0.001	0.921 (0.879, 0.965)

VVS, vasovagal syncope.

## Data Availability

The raw data supporting the conclusions of this article will be made available by the authors upon request due to privacy reasons.

## Abbreviations

The following abbreviations are used in this manuscript:

BMI	Body mass index
DBP	Diastolic blood pressure
FMD	Flow-mediated vasodilation
HF	High frequency
HR	Heart rate
HRV	Heart rate variability
HUTT	Head-up tilt test
LF	Low frequency
pNN50	Percentage of adjacent normal-to-normal interval differences greater than 50 ms
rMSSD	Root mean square of successive differences between adjacent normal cycles
SBP	Systolic blood pressure
SDANN	Standard deviation of the 5 min average normal-to-normal intervals
SDNN	Standard deviation of all normal-to-normal intervals
VIF	Variance inflation factor
VVS	Vasovagal syncope
